# Supplementary material for: Safety of Anti-Angiogenic Drugs in Pediatric Patients with Solid Tumors: A Systematic Review and Meta-Analysis
Source: Cancers (Basel). 2022 Oct 28;14(21):5315. doi: 10.3390/cancers14215315 (PMC9654149; doi:10.3390/cancers14215315)
Supplement: Supplementary file 1 [file cancers-14-05315-s001.zip › cancers-1966529-supplementary.pdf]

Supplementary materials

Table S1. Search string for PubMed, ISI Web of Science and Clinicaltrials.gov

**PUBMED**
((anti-VEGF or "antiangiogenic drug" or bevacizumab or sorafenib or sunitinib or cabozantinib or axitinib or aflibercept or dovitinib or lenvatininb or nintedanib or pazopanib or ponatinib or ramucirumab or regorafenib or rivoceranib or tivozatinib or trebananib or vandetanib) AND (Pediatric or Paediatric or Child or Childhood or Children or "young adults")) AND (Tumor or cancer or tumors or cancers or carcinoma) Filters: Clinical Study, Clinical Trial, Phase I, Phase II, Clinical Trial, Phase III, Clinical Trial, Phase IV, Observational Study<br><br>Date: 2022/3/31
**ISI WEB OF KNOWLEDGE**
anti-VEGF or “antiangiogenic drug” or bevacizumab or sorafenib or sunitinib or cabozantinib or axitinib or aflibercept or dovitinib or lenvatinib or nintedanib or pazopanib or ponatinib or ramucirumab or regorafenib or rivoceranib or tivozanib or trebananib or vandetanib (All Fields) and Pediatric or Paediatric or Child or Childhood or Children or “young adults” (All Fields) and Tumor or cancer or tumors or cancers (All Fields) and "clinical study" or "observational study" or "clinical trial" or "phase I" or "phase II" or "phase III" (All Fields)
**CLINICALTRIALS.GOV**
Condition or disease: cancer or tumor or carcinoma<br>Other term: bevacizumab or sorafenib or sunitinib or cabozantinib or axitinib or aflibercept or dovitinib or lenvatininb or nintedanib or pazopanib or ponatinib or ramucirumab or regorafenib or rivoceranib or tivozatinib or trebananib or vandetanib<br>Filter: Child (birth-17)

Table S2. Characteristic of the safety population of the included studies.

Reference	NCT number	Considered for severity (yes/no)	Safety population severity	Female*	Median age, years (range or inclusion criteria)*	Incomplete or selective report of AE (Severity)	Note for meta-analysis	Considered for seriousness (yes/no)	Safety population seriousness	Female*	Median age, years (range or inclusion criteria)*	Incomplete or selective report of AE (Seriousness)	Note
Glade Bender 2008	-	Yes	19	ns	ns (<21)	Threshold 10%		No					
Gorsi 2018	-	Yes	15	10	7 (1-20)	Grade 1 missing		No					
Okada 2013	-	Yes	11	6	9 (3-22)	Unclear if some Grade 3 AEs were reported (method section)		No					
Fangusaro 2013	NCT00381797	Yes	92	ns	ns (<20)	No selective report of AEs	Probably underestimation of events both for grade 1-2 and 3-4 (higher number between grade 1 - 2 and 3-4 was extracted respectively).	Yes	92	ns	ns (<20)	-	
Couec 2012	-	Yes	28	ns	11 (1-20)	Two events of wound healing were considered as grade 1-2 despite grade was not reported		No					
Kalra 2015	-	Yes	16	7	8.6 (2-15)	Grade 1 missing; Nausea vomiting not distinguished	Probable underestimation of grade 1-2	No					
De Marcellus 2022	-	Yes	72	ns	7.8 (1-19)			No					
Modak 2017	NCT01114555	Yes	33	8	6.4 (2-26)			Yes	34	8	6 (1-33)	-	
Levy 2020	NCT01217437	Yes	52	16	10 (0-18)	Grade 1-2 missing	No possible use for Grade 1-2	Yes	52	16	10 (0-18)	-	
Hummel 2016	NCT00890786	Yes	27	14	10 (3-29)	Grade 1 missing; threshold 10%	Probable underestimation of grade 1-2	No					
Schiavetti 2019	-	Yes	12	3	11 (0-29)			No					
Aguilera 2013	-	Yes	9	3	4 (2-7)	Grade 1 missing	Probable underestimation of grade 1-2	No					
Wagner 2013	NCT00786669	Yes	13	5	12 (1-22)	Grade 1-2 missing	No possible use for Grade 1-2	No					
Venkatramani 2013	NCT00993044	Yes	12	4	11 (4-19)	Grade 1 missing; Grade 2 reported only for hematological	Probable underestimation of grade 1-2;	Yes	12	4	11 (4-19)	Threshold 5% (non-serious)	
El-Khouly 2021	-	Yes	9	4	7.7 (5-15)			No					
Mascarenhas 2019	NCT01222715	Yes	42	ns	ns (<29)	No grade 1-2: grade 3-4 threshold 10%	No possible use for Grade 1-2	Yes	44	22	Ns (<29)	-	
Navid 2017	NCT00667342	Yes	31	15	12.8 (7-20)	Only few grade 1-2 reported	Probable underestimation of grade 3-4	Yes	42	ns	ns	Threshold 5% (non-serious)	

Chisholm 2017	NCT00643565	Yes	71	ns	ns (<18)	Grade 1-2 missing	No possible use for Grade 1-2	Yes	71	ns	ns (<18)	Threshold 5% (non-serious)	
Millan 2016	-	Yes	16	9	2.8 (0-4)			No					
Piha-Paul 2014	NCT00610493	Yes	6	ns	6 (3-14)			No					
Federico 2020	NCT00665990	Yes	24	ns	ns (<22)	Grade 1 missing; Threshold 10%	Probable underestimation of grade 1-2;	No					
Su 2020	NCT00879437	Yes	38	18	7.9 (3-20)	No grade 1 reported	Probable underestimation of grade 1-2; Missing hematological grade 1-2	Yes	38	18	7.9 (3-20)	Threshold 5% (non-serious)	
Santana 2020	NCT00756340	Yes	15	8	11.8 (5-18)	Grade 1-2 missing	No possible use for Grade 1-2	No					
Reismuller 2010	-	Yes	30	11	9.9 (1.5-18)			No					
Widemann 2012	NCT01445080	Yes	30	ns	Ns (≤21)	Threshold 10%		Yes	36	18	Ns (6-21)		Selected only those with solid tumors (part A)
Karajannis 2014	NCT01338857	Yes	12	6	9.2 (3-15)			Yes	12	6	9.2 (3-15)	Threshold 5% (non-serious)	
Meany 2021	NCT01518413	Yes	15	6	14.4 (5-20)	Missing grade 1 cycle		No					
Keino 2020	-	Yes	6	2	8.7 (6-16)	Only toxicities after first cycle		No					
Reed 2016	NCT01683149	Yes	12	4	13 (8-18)	Grade 1-2 missing	No possible use for Grade 1-2	No					
DuBois 2011	NCT00387920	Yes	21	12	13.9 (4-20)	Threshold 10% (cycle 1); two missed patients for evaluation of hematological toxicities		No					
Wetmore 2016	NCT01462695	Yes	29	11	13.4 (3-20)			Yes	29	11	13.4 (3-20)		
Verschuur 2019	NCT01396148	Yes	6	5	14 (13-16)	AEs occurred in at least 2 patients out of 6		No					
Glade Bender 2013	NCT00929903	Yes	51	25	12.9 (4-24)	Threshold 10%		No					
Weiss 2020	NCT02180867	Yes	11	ns	Ns (≤18)	Grade 1-2 missing; Only hematological AEs reported	No possible use for Grade 1-2	Yes	32	ns	Ns (≤18)	No total number of pediatric patients with at least one serious non serious AEs	Number of pediatric patients specified
Russo 2020	-	Yes	17	9	14 (5-19)			No					
Broniscer 2010	NCT00472017	Yes	35	20	6.4 (3-16)	Not reported hypertension grade 1; For further courses no number of cycles		No					
Broniscer 2013	NCT00996723	Yes	25	13	5.8 (2-17)	Not reported hypertension grade 1; For further courses no number of cycles		No					

Kraft 2018	NCT00514046	Yes	17	9	13 (9-17)	Missing grade 1; Some reactions could not be graded	Possible underestimation of grade 1-2	Yes	17	9	13 (9-17)		
Geoerger 2021	NCT02085148	Yes	41	21	13.0 (3-17)	Threshold 20%; Only treatment emergent adverse events		No					
Leary 2017	NCT01538095	Yes	19	ns	12.1 (2-21)	Threshold 10%		No					
Chuk 2018	NCT01709435	Yes	41	20	13 (4-18)		Possible underestimation of grade 1-2 and 3-4	No					
Geller 2018	NCT02164838	Yes	18	8	13.5 (5-17)	Threshold 10% (Non-hematological toxicities)		No					
Glade Bender 2012	NCT00622414	Yes	21	10	12.9 (1.9-21.6)	Threshold 10% (Non-hematological toxicities)	Possible underestimation of grade 1-2	No					
Gaspar 2021 (I)	NCT02432274	Yes	54 (23,31)	11, 18	12 (13-17), 15 (9-22)	Threshold 10%; Reported Treatment emergent adverse events		Yes	55	Ns	Ns (<25)	Threshold 5% (non-serious)	Cohort 1, 2A and 2B
Gaspar 2021 (II)	NCT02432274	Yes	35	12	15 (13-17)	Threshold 10% for grade 1–2; Reported Treatment emergent adverse events		Yes	42 subjects (22 in cohort 3A + 20 in cohort 3B)	ns	Ns (<25)	Threshold 5% (non-serious)	Combination cohorts 3A-3B as in the study
De Wire 2015	NCT00883688	No					Number of episodes and not patients experiencing AE	Yes	24	7	10 (2-21)		
Grill 2018	NCT01390948	No					No specific AEs	Yes	60	ns	Ns (<17)	Threshold 5% (non-serious)	
Metts 2022	NCT00876993	No					No number of patients but only cycles – not possible to calculate incidence	Yes	26	8	12 (3-22)		
-	NCT01956669	No						Yes	57	24	ns	Threshold 5% (non-serious)	
-	NCT01236560	No						Yes	32	12	11.8 (3-20)		Arm III
-	NCT01201850	No						Yes	7	2	Ns (<26)	Threshold 5% (non-serious)	
Glod 2019	NCT02015065	No					No safety per pediatric patients but in the overall cohort	Yes	2	2	Ns (<18)		
-	NCT01492673	No						Yes	9	6	12 (8-13)	Threshold 5% (non-serious)	
-	NCT00516295	No						Yes	6	ns	15 (12-20)		
-	NCT01502410	No						Yes	10	9	11 (5-21)		
-	NCT02564198	No						Yes	29	12	Ns	Threshold 5% (non-serious)	

*referred to patients in which safety was evaluatated

Table S3. Cochrane risk of bias tool assessment for randomized controlled studies

REF	NVT	Random sequence generation	Allocation concealment	Blinding of participants and personnel	Blinding of outcome assessment	Incomplete outcome data	Selective reporting	Overall risk
Levy 2020	NCT01217437	Unclear	Unclear	High risk	High risk	Low risk	Low risk	High risk
Grill 2018	NCT01390948	Low risk	Unclear	High risk	High risk	Low risk	Unclear	High risk
-	NCT01236560	Unclear	Unclear	High risk	High risk	Low risk	Unclear	High risk
-	NCT00516295	High risk	Unclear	High risk	High risk	High risk	Unclear	High risk
Mascarenhas 2019	NCT01222715	Unclear	Unclear	High risk	High risk	Low risk	Unclear	High risk
Weiss 2020	NCT02180867	Low risk	Low risk	High risk	High risk	Low risk	Unclear	High risk
Chisholm 2017	NCT00643565	Low risk	Unclear	High risk	High risk	Low risk	Unclear	High risk

Green: Low risk; Yellow: Unclear; Red: High risk

Table S4. MINORS tool assessment

REF	NCT number	A clearly stated aim	Inclusion of consecutive patients (exclusion)	Prospective collection of data (protocol)	Endpoints appropriate to the aim of the study	Unbiased assessment of the study endpoint (blind)	Follow-up period appropriate to the aim of the study	Loss to follow up less than 5%	Prospective calculation of study size	Total score /16
Glade Bender 2008	-	2	2	2	2	0	0	1	0	9/16
Gorsi 2018	-	0	0	2	0	0	0	2	0	4/16
-	NCT01201850	2	2	2	2	0	0	2	0	10/16
Okada 2013	-	2	2	2	2	0	0	1	0	9/16
Fangusaro 2013	NCT00381797	2	2	2	2	0	0	1	2	11/16
Modak 2017	NCT01114555	2	2	2	2	0	2	2	2	14/16
Hummel 2016	NCT00890786	2	2	2	2	0	2	2	2	14/16
Metts 2022	NCT00876993	2	2	2	2	0	0	2	0	10/16
Schiavetti 2019	-	2	2	0	2	0	2	2	2	12/16
Wagner 2013	NCT00786669	2	2	2	2	0	0	2	0	10/16
Venkatramani 2013	NCT00993044	2	2	2	2	0	0	1	0	9/16
El-Khouly 2021	-	2	2	2	2	0	2	2	0	12/16
-	NCT01492673	2	2	2	2	0	2	2	0	12/16
Navid 2017	NCT00667342	2	2	2	2	0	2	2	2	14/16
Piha-Paul 2014	NCT00610493	2	2	2	2	0	0	2	0	10/16
Federico 2020	NCT00665990	2	2	2	2	0	0	2	2	12/16
Su 2020	NCT00879437	2	2	2	2	0	0	2	2	12/16
Santana 2020	NCT00756340	2	2	2	2	0	0	1	2	11/16
De Wire 2015	NCT00883688	2	2	2	2	0	0	2	2	12/16
Widemann 2012	NCT01445080	2	2	2	2	0	0	2	0	10/16
Karajannis 2014	NCT01338857	2	2	2	2	0	0	2	2	12/16
-	NCT01502410	2	2	2	2	0	0	2	0	10/16
Meany 2021	NCT01518413	2	2	2	2	0	0	0	0	8/16
Keino 2020	-	2	0	0	2	0	2	2	0	8/16
Reed 2016	NCT01683149	2	2	2	2	0	0	1	0	9/16
DuBois 2011	NCT00387920	2	2	2	2	0	0	2	0	10/16
Wetmore 2016	NCT01462695	2	2	2	2	0	2	2	2	14/16
Verschuur 2019	NCT01396148	1	2	2	1	0	2	0	2	10/16

Glade Bender 2013	NCT00929903	2	2	2	2	0	0	2	0	10/16
-	NCT01956669	2	2	2	2	0	0	0	0	8/16
Broniscer 2010	NCT00472017	2	0	2	2	0	0	2	0	8/16
Broniscer 2013	NCT00996723	2	2	2	2	0	0	2	0	10/16
Kraft 2018	NCT00514046	2	2	2	2	0	2	2	2	14/16
Glod 2019	NCT02015065	2	2	2	2	0	0	2	2	12/16
Geoerger 2021	NCT02085148	2	2	2	2	0	2	2	2	14/16
-	NCT02564198	2	2	2	2	0	0	0	2	10/16
Leary 2017	NCT01538095	2	2	2	2	0	0	0	0	8/16
Chuk 2018	NCT01709435	2	2	2	2	0	0	0	0	8/16
Geller 2018	NCT02164838	2	2	2	2	0	0	1	2	11/16
Glade Bender 2012	NCT00622414	2	2	2	2	0	0	2	0	10/16
Gaspar 2021 (I) ALONE	NCT02432274	2	2	2	2	0	2	0	2	12/16
Gaspar 2021 (II)	NCT02432274	2	2	2	2	0	2	2	2	14/16

Green: good quality; Yellow: Moderate quality; Red: Poor quality

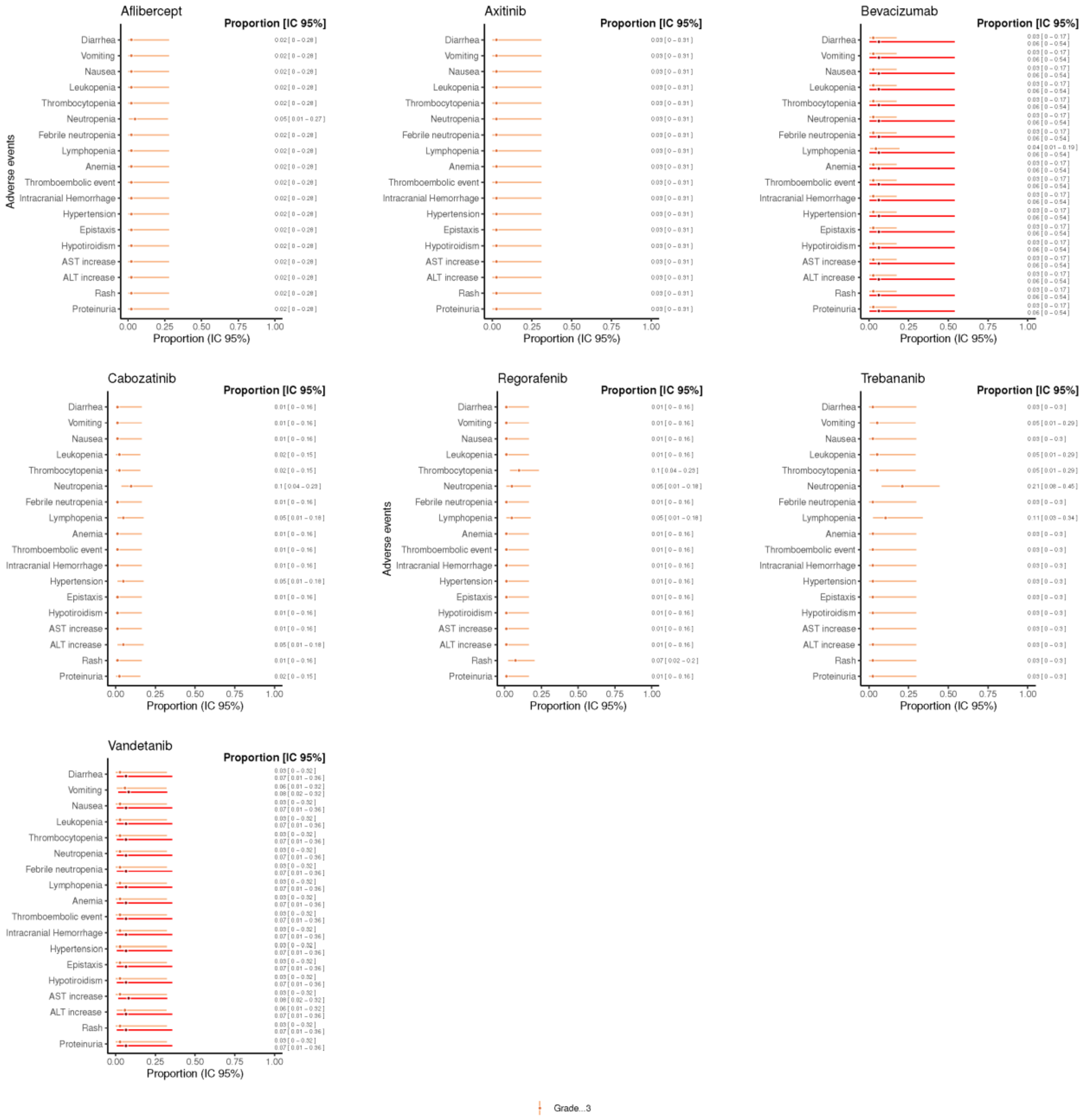

Figure S1. Proportion of serious/Grade ≥3 AEs for pediatric patients receiving antiangiogenic drugs as monotherapies (drugs assessed in less than 20 patients or not reporting both serious/Grade ≥3 AEs)

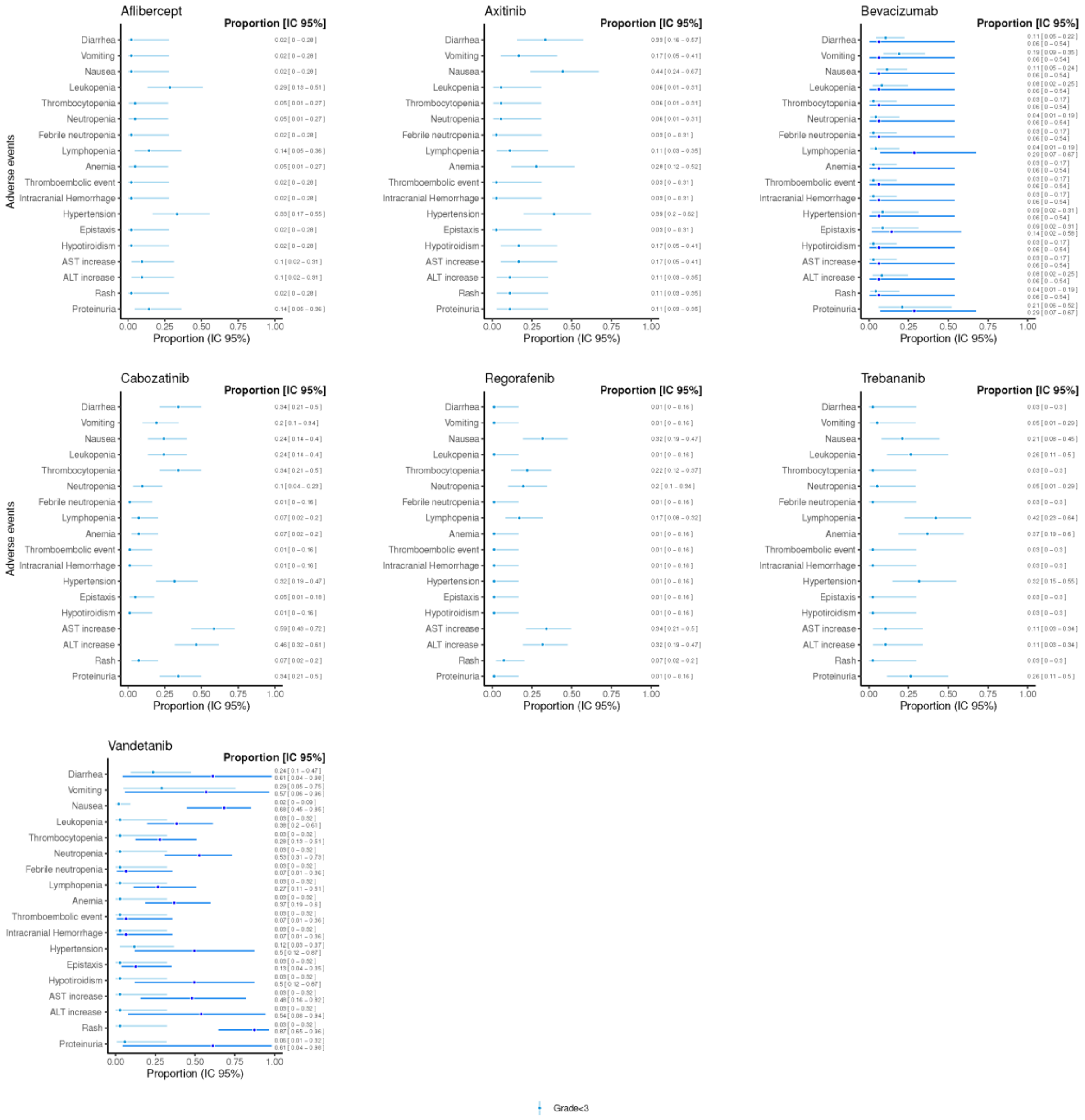

Figure S2. proportion of non-serious/Grade <3 AEs for pediatric patients receiving antiangiogenic drugs as monotherapies (drugs assessed in less than 20 patients or not reporting both non-serious/Grade <3 AEs)

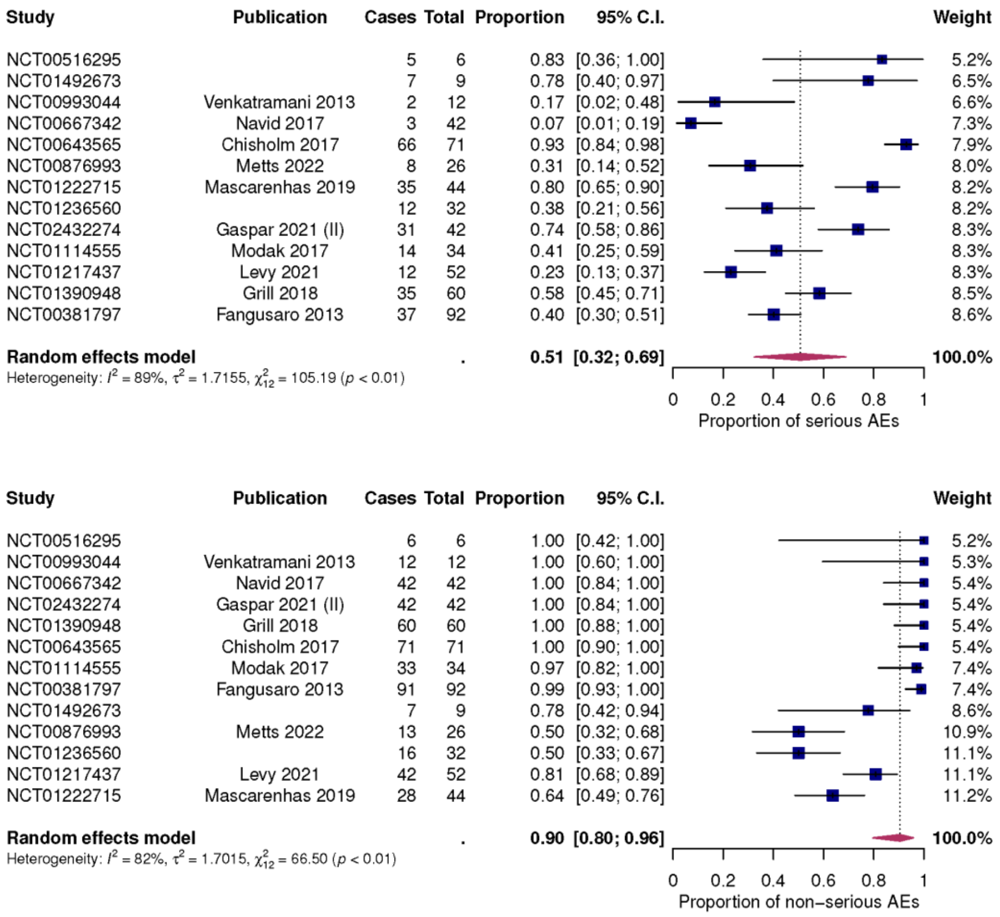

Figure S3. Overall proportion of serious (Panel A) and non-serious AEs (Panel B) for bevacizumab and lenvatinib plus chemotherapy (no strata by chemotherapy combinations)
